# Sarajevo Declaration on Integrity and Visibility of Scholarly Publications

**DOI:** 10.3325/cmj.2016.57.527

**Published:** 2016-12

**Authors:** Izet Mašić, Edin Begić, Doncho M. Donev, Srećko Gajović, Armen Yuri Gasparyan, Miro Jakovljević, Dejan B. Milošević, Osman Sinanović, Šekib Sokolović, Selma Uzunović, Enver Zerem

**Affiliations:** 1 Faculty of Medicine, University of Sarajevo, Sarajevo, Bosnia and Herzegovina; 2 Academy of Medical Sciences of Bosnia and Herzegovina, Sarajevo, Bosnia and Herzegovina; https://orcid.org/0000-0002-9080-5456; 3 Faculty of Medicine, Sarajevo School of Science and Technology, Sarajevo, Bosnia and Herzegovina; 4 Health Care Centre, Maglaj, Bosnia and Herzegovina; http://orcid.org/0000-0001-6842-262X; 5 Institute of Social Medicine, Faculty of Medicine, University of “Ss. Cyril and Methodius” in Skopje, Skopje, Republic of Macedonia; https://orcid.org/0000-0002-5237-443X; 6 Croatian Institute for Brain Research, University of Zagreb School of Medicine, Zagreb, Croatia; http://orcid.org/0000-0001-8668-5239; 7 Departments of Rheumatology and Research & Development, Dudley Group NHS Foundation Trust (Teaching Trust of the University of Birmingham, UK), Russells Hall Hospital, Dudley, West Midlands, UK; https://orcid.org/0000-0001-8749-6018 a.gasparyan@gmail.com; 8 Department of Psychiatry, University Hospital Centre Zagreb, Zagreb, Croatia; 9 Faculty of Science, University of Sarajevo, Sarajevo, Bosnia and Herzegovina; 10 Academy of Sciences and Arts of Bosnia and Herzegovina, Sarajevo, Bosnia and Herzegovina; http://orcid.org/0000-0001-5060-3318; 11 Department of Neurology, University Clinical Center Tuzla, Faculty of Medicine, University of Tuzla, Tuzla, Bosnia and Herzegovina; http://orcid.org/0000-0001-8957-7284; 12 Institute for Heart Diseases, University Clinical Center Sarajevo, Sarajevo, Bosnia and Herzegovina; http://orcid.org/0000-0001-6321-4186; 13 Department for Laboratory Diagnostics, Institute for Public Health and Food Safety, Zenica, Bosnia and Herzegovina; http://www.orcid.org/0000-0003- 1852-1572; 14 Department of Medical Sciences, Academy of Sciences and Arts, Sarajevo, Bosnia and Herzegovina; 15 Department of Gastroenterology and Hepatology, University Clinical Center Tuzla, Tuzla, Bosnia and Herzegovina; http://orcid.org/0000-0001-6906-3630

## Preamble

Non-mainstream scholarly journals are struggling to maintain a healthy flow of the best submissions, influence science growth locally and internationally, and become indexed in prestigious bibliographic databases. There are financial and non-financial factors confounding editing and publishing practices across low-resource countries. Publishers in these countries often face difficulties with involving experienced reviewers and editors in the processing of submissions to their journal. As a result, non-mainstream scholarly journals are at risk of publishing unchecked, poorly edited, erroneous, and unethical papers. They face challenges of inappropriate authorship, non-disclosure of conflicts of interests, plagiarism, and other forms of scientific misconduct. Journals’ applications to global indexing abstract and citation databases are often declined due to the lack of transparent and ethical editorial strategies, inappropriate scope of interest, low level of evidence, and low citation rates. A hhandful of the indexed regional journals are aggressively targeted by unethical authors and brokering editing agencies, exploiting the deficiencies in the editorial strategies and thus further damaging the reputation of the journals. To curb the problems with editing and publishing, journal editors from non-mainstream science countries are encouraged to upgrade their strategies of ethical editing and research reporting in accordance with the updated recommendations of editorial associations, such as the International Committee of Medical Journal Editors (ICMJE), the Committee on Publication Ethics (COPE), and the Council of Science Editors (CSE). Academic institutions and professional societies from these countries are called to provide guidance for researchers and science editors and allocate resources for improving editing and publishing practices.

How to address the problems with journal publishing in non-mainstream science countries was discussed at the First Mediterranean Seminar on Science Writing, Editing and Publishing, which was organized by the Academy of Medical Sciences of Bosnia and Herzegovina in Sarajevo, December 2-3, 2016. The idea of organizing such a meeting and drafting an instructive document for editors and publishers struggling with poor quality and visibility of their journals was proposed by numerous regional experts. They voiced their concerns and suggested to act jointly. The Seminar attracted more than 100 researchers, experienced journal editors, and publishers from Balkan and Mediterranean countries, who shared their experience with writing, reviewing, editing, and publishing. Updates to the recommendations of the most influential editorial associations, such as the ICMJE, COPE, and CSE, were presented to incorporate in the regional journal instructions. Participants had a unique opportunity to receive hands-on training on editing from the flagship regional journals, such as the *Croatian Medical Journal* and other MEDLINE-indexed journals. As the main problems with editing regional journals were highlighted, the decision was made to draft this Declaration. Journal editors from non-mainstream science countries are called to upgrade and enforce their instructions for authors in accordance with the listed points on integrity and visibility.

## Aim

The Sarajevo Declaration is aimed at upgrading standards of editing and publishing scholarly journals across Balkan and Mediterranean countries. Journal editors from regional and other non-mainstream science countries are encouraged to familiarize themselves with the statements and amend their instructions accordingly.

## Expected outcomes

The endorsement and enforcement of the Sarajevo Declaration may help avoid ‘wasteful’ or unethical publishing practices and improve visibility, scientific prestige, and indexability of the adherent scholarly publications.

**Strategic points for action**
- Transparency of in-house editorial procedures and external editing support
- Support of professional editorial teams
- Focus on regional and local scientific research problems defined in the journal aims and scope
- Promotion of ethical research and reviews

## Statements

1. **Scholarly publications** are essential for research productivity, academic promotion, sharing professional information, and networking among scientists worldwide. Authors, reviewers, and editors are required to ensure the trustworthiness and ethical soundness of what they write, edit, and publish. To maintain the quality and ensure the impact of their publications, all stakeholders in science communication should make effort to ensure the integrity and promote innovative and evidence-based sources of information.

2. **Scholarly papers** are final products of collective efforts of all stakeholders in science communication. It is increasingly important to promote these papers post-publication by indexing and archiving on relevant global digital platforms. Responsible editors and publishers alike are in the position to contribute to the post-publication communication. To improve visibility of their publications, authors can rely on reliable social media, sharing platforms, and individual and institutional repositories.

3. **Authors, reviewers, and editorial board members** can increase visibility of their scholarly activities by registering with the Open Researcher and Contributor ID (ORCID) and providing information on their own authoring, reviewing, and publishing activities via their permanent accounts. Publishers of scholarly journals can maintain the integrity of pre- and post-publication communication by joining the ORCID global initiative.

4. **Erroneous publications** are common, and take place because of the authors’, editors’, and publishers’ oversights. Authors, reviewers, editors, and readers have the responsibility to notify publishers about any instances of known research misconduct and erroneous publications, necessitating corrections or retractions.

5. **Effective functioning of scholarly journals** is dependent on skilled individuals involved in the processing of manuscripts. Publishers encountering problems with erroneous and poorly written publications should expand their editorial teams by inviting experienced editors responsible for statistics, publication ethics, language, and design.

6. **Continuing professional development** (CPD) should be complemented by publications in scholarly journals that serve as platforms for distributing professional information of interest to both novice and seasoned authors. To achieve this goal, publishers may create journal sections for students, researchers, and specialists seeking CPD credits. Efforts should be made by publishers to acknowledge their contributors and obtain CPD credits for publishing and reviewing activities.

7. **Professional societies** can take the lead and contribute to the promotion of scholarly journals by taking responsibility for healthy flow of journal submissions from their memberships, quality checks, and publishing established and start-up periodicals and awarding the contributors with academic credits.

8. **Science editors** should adhere to the most recent recommendations of global editorial associations and incorporate relevant sections in their journal’s instructions to improve the quality of pre- and post-publication communication.

9. **Websites and editorial management platforms** of scholarly journals should contain transparent information on the editorial management, peer review, open access or subscription models, and acceptable editing practices. Commercial editing services, which are offered by publishers and other organizations, may help improve the quality of journal submissions. However, all these services require transparency and acknowledgment in accordance with the recommendations of global editorial associations.

10. **Traditional and alternative impact indicators** are instrumental for assessing the scholarly journals in terms of distributing information, attracting readership, and facilitating science growth. Combined quantitative and qualitative approach to citations, downloads, and distributions of individual papers through social networking channels can reveal interest of readers toward certain topics and types of publication, but not necessarily the quality of scholarly journals. Editors and publishers can increase the impact of their journals by improving the functionality of their journal websites and online content, ensuring the completeness of meta-data in the published papers.

